# Remarkable changes in the choice of timing to discuss organ donation with the relatives of a patient: a study in 228 organ donations in 20 years

**DOI:** 10.1186/cc10481

**Published:** 2011-10-07

**Authors:** Yorick J de Groot, Hester F Lingsma, Mathieu van der Jagt, Jan Bakker, Jan NM IJzermans, Erwin JO Kompanje

**Affiliations:** 1 Department of Intensive Care, Erasmus MC University Medical Center, PO Box 2040, 3000 CA, Rotterdam, The Netherlands; 2 Department of Public Health, Center for Medical Decision Making, Erasmus MC University Medical Center, PO Box 2040, 3000 CA, Rotterdam, The Netherlands; 3 Department of Surgery, Erasmus MC University Medical Center, PO Box 2040, 3000 CA, Rotterdam, The Netherlands

## Abstract

**Introduction:**

We studied whether the choice of timing of discussing organ donation for the first time with the relatives of a patient with catastrophic brain injury in The Netherlands has changed over time and explored its possible consequences. Second, we investigated how thorough the process of brain death determination was over time by studying the number of medical specialists involved. And we studied the possible influence of the Donor Register on the consent rate.

**Methods:**

We performed a retrospective chart review of all effectuated brain dead organ donors between 1987 and 2009 in one Dutch university hospital with a large neurosurgical serving area.

**Results:**

A total of 271 medical charts were collected, of which 228 brain dead patients were included. In the first period, organ donation was discussed for the first time after brain death determination (87%). In 13% of the cases, the issue of organ donation was raised before the first EEG. After 1998, we observed a shift in this practice. Discussing organ donation for the first time after brain death determination occurred in only 18% of the cases. In 58% of the cases, the issue of organ donation was discussed before the first EEG but after confirming the absence of all brain stem reflexes, and in 24% of the cases, the issue of organ donation was discussed after the prognosis was deemed catastrophic but before a neurologist or neurosurgeon assessed and determined the absence of all brain stem reflexes as required by the Dutch brain death determination protocol.

**Conclusions:**

The phases in the process of brain death determination and the time at which organ donation is first discussed with relatives have changed over time. Possible causes of this change are the introduction of the Donor Register, the reintroduction of donation after circulatory death and other logistical factors. It is unclear whether the observed shift contributed to the high refusal rate in The Netherlands and the increase in family refusal in our hospital in the second studied period. Taking published literature on this subject into account, it is possible that this may have a counterproductive effect.

## Introduction

### Background

In The Netherlands, the concept of brain death was accepted in the 1970s [1-4]. In addition, in 1996, the Dutch Organ Donation Act (DODA) [5] became effective. The DODA was drawn up with four objectives in mind: (a) to clarify the legal position of organ donation, (b) to increase the supply of organs and tissues, (c) to ensure the fair distribution of organs and tissues and (d) to prevent trade in organs and tissues. In line with the DODA, in 1997 the Dutch government legally established the brain death criteria described by the Dutch Health Council.

A second initiative integrated with the DODA was the establishment of a national Donor Register (DR) in 1998. The DR, designed as an opt-in system, allows people to register their preferences regarding organ, bone and tissue donation, including their refusal to donate. Those who are not registered in the DR can donate with explicit consent from the patient's next of kin [5].

If the patient has registered his or her consent or objection, the physician is expected to inform the family about the patient's wishes and explain the steps involved in the donation process if applicable. Consent given by the patient in the DR for organ donation permits the physician to begin organ-preserving treatment. Before 1998, individuals in The Netherlands could register their will concerning organ donation in a handwritten donor card. A third development in the 1990s was reintroduction of donation after circulatory death (DCD) in The Netherlands.

After the establishment of the brain death criteria, brain dead (BD) donors became the most important source of organs due to the superior organ quality and because these donors are the only source of hearts for transplantation. However, due to the declining availability of BD donors in The Netherlands, DCD became a reasonable and necessary alternative and was, therefore, reintroduced, first only for allocation of kidneys, and later for liver and lungs. In the past 15 years in The Netherlands, there has been a decline of donation after brain death (DBD) from 915 donors, 88.6% of the total number of donors, to 697 donors (58.4%), whereas DCD increased accordingly from 118 donors (11.4%) to 453 donors (41.6%). The decline in the number of DBD is completely compensated by an increase in the number of DCD [6]. This trend is consistent with literature from outside of Europe [7]. Due to the nature of DCD, permission for organ donation is requested prior to patient death. This sequence of consent is in contrast with obtaining consent for DBD, which is founded in the brain death protocol, in which consent can only be obtained after the formal determination of brain death [5]. Some believe that the issue of organ donation in case of a catastrophic brain injury is nowadays discussed with relatives earlier in the process (before formal determination of brain death) than it was before the year 2000. This may have a negative effect on the consent rate.

### Study aims

We aimed to study whether the choice of timing of discussing organ donation for the first time with the relatives of a patient with catastrophic brain injury in The Netherlands has changed through time and to explore the possible consequences of such a change. Secondly, we investigated how thoroughly the process of brain death determination was performed over time by studying the number of medical specialists involved. Thirdly, we studied the possible influence of a hand-written donor card (before 1998) and registration in the DR (after 1998) on consent rate.

## Materials and methods

We conducted a retrospective chart review of all patients who became brain dead during their stay in the intensive care unit of the Erasmus MC University Medical Center between 1987 and 2009, and of who donated organs for transplantation. This study was exempt from review and approval by the institutional Ethics Committee due to the retrospective, observational nature. We obtained a list of patients from the in-house transplant coordinator and crosschecked this with the central hospital patient registry. The following data were extracted: patient age, sex, cause of death, the moment of neurological examination of brain stem reflexes, the moment of confirmatory testing for brain death, the time at which organ donation was first discussed with the relatives of the patient, permission for organ donation by family or patient as documented in the patient's medical chart, the time at which consent was requested during the process and the number and speciality of the reviewing independent medical specialists. Exclusion criteria were age < 12 years, insufficient data or failure to retrieve the medical chart from the hospital archive and BD patients who were converted to DCD. The age limit of 12 years was selected because in The Netherlands an individual may only register his or her will in the DR after this age.

### Timing of discussing organ donation with the relatives

To analyse the time at which organ donation was first discussed with the relatives of the patient, we identified three events surrounding the process of determination of brain death:

1. The determination of the absence of consciousness (Glasgow Coma Score of 3), the absence of all brain stem reflexes as assessed by a neurologist or neurosurgeon as described by the Dutch brain death protocol [4].

2. Performing the confirmatory tests. In The Netherlands, an electroencephalogram (EEG) followed by an apnea test is mandatory to declare brain death and to proceed with organ donation [4].

3. The time at which organ donation was first discussed with the relatives of the patient in relation to the first two events or the time at which consent for the organ donation procedure was obtained from the next of kin, or in the case of consent by a patient in the DR, assent of the next of kin.

**Figure 1:**
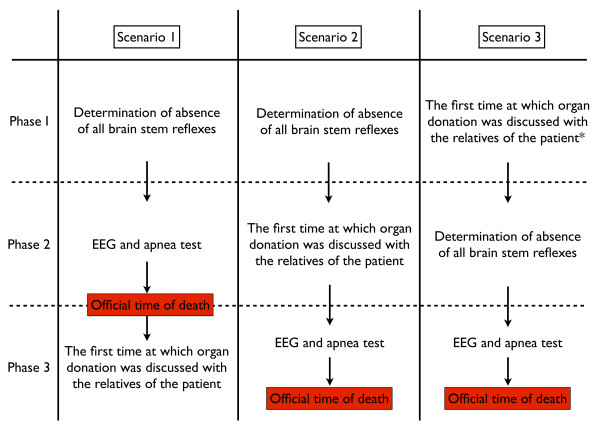
**Flowchart of three possible scenarios concerning the moment of requesting organ donation**. (* patients who were regarded as potential brain dead organ donors and for whom further treatment was deemed futile).

We determined three possible scenarios for the process of brain death determination and the time at which the issue of organ donation was first discussed with the relatives of a BD patient (Figure 1), which could lead to consent or objection to organ donation. The ICU physician approaches the family with the issue of organ donation. Consent for organ donation by the relatives in The Netherlands is a verbal agreement between the ICU physician and the relatives of the patient. This is not further formalised by a signed agreement by the relatives. The decision about organ donation by the relatives or the patient is documented in the patient's medical chart, which, under Dutch law, serves as a legal document.

### Consultation by independent medical experts

We investigated how thoroughly the process of brain death determination was performed over time by studying the number of independent medical specialists involved by reviewing the medical charts on written statements of brain death confirmation.

### Influence of hand-written donor card and registration in donor register on consent rate

We studied the possible influence of the DR on the consent rate by searching for documentation in the medical charts on the presence of a written donor card or print-out of the DR.

Because changes in legislation were introduced in 1998 in The Netherlands, we divided the study cohort into two periods to study the changes over time. Period 1 consisted of the patients who died between 1987 and 1998, and period 2 consisted of the patients who died between 1999 and 2009.

### Statistics

Normally distributed continuous variables are described using their means and standard deviations. Skewed continuous variables are described using medians and inter-quartile ranges. Binary variables are described using proportions. Differences between the two subgroups were tested using Student's *t*-test for normally distributed continuous variables, and the Mann-Whitney U test was used for skewed continuous variables. All binary variables were analysed using the Chi-square test.

## Results

Between 1987 and 2009, 271 patients were declared brain dead and donated one or more organs; 19 patients in this cohort were excluded due to the fact that relevant data for analysis could not be extracted from the medical chart, and 24 patients younger than 12 years were excluded due to the minimal age requirement. Therefore, 228 patients were included in the study for further analysis. In this cohort, the most frequent fatal conditions of effectuated brain-dead organ donors were subarachnoid haemorrhage (SAH) (50.0%), traumatic brain injury (TBI) (28.9%) and intracerebral haemorrhage (ICH) (12.7%) (Table 1).

**Table 1:** Causes of brain death in 228 effectuated organ donors age ≥ 12 yrs (1987 to 2009) *n *= 228

Cause n (%)	1987 to 1998	1999 to 2009	Total	*P*-value
SAH	51 (41.5)	63 (60.0)	114 (50.0)	0.049
ICH	18 (14.6)	11 (10.5)	29 (12.7)	
TBI	44 (35.8)	22 (21.0)	66 (28.9)	
Other	10 (8.1)	9 (8.5)	19 (8.4)	
Total	123	105	228	

ICH, intracerebral haemorrhage; SAH, subarachnoid haemorrhage; TBI, traumatic brain injury

### Timing of discussing organ donation with the relatives

**Figure 2:**
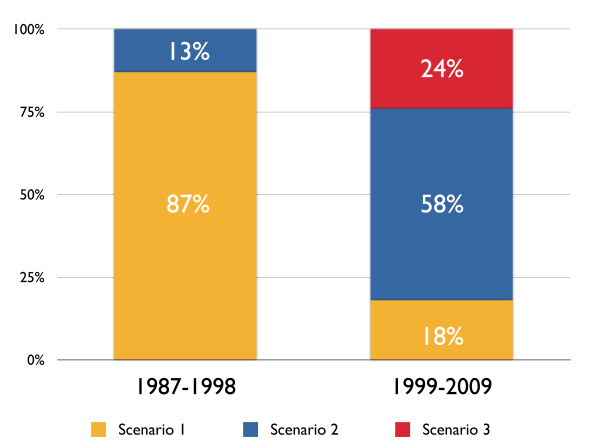
**Sequence of brain death determination**.

Figure 2 shows the sequence of events surrounding the formal determination of brain death and the timing of the first discussion of organ donation with the patients' relatives. In the first period, Scenario 1 was the most common practise, occurring in 87% of the cases. In 13% of the cases, the issue of organ donation was raised before the first EEG (Scenario 2, Figure 2). After 1998, we observed a shift in this practise. Scenario 1 occurred in only 18% of the cases. In 58% of the cases, the issue of organ donation was discussed before the first EEG, but after confirming the absence of all brain stem reflexes (Scenario 2), and in 24% of the cases, the issue of organ donation was discussed after the prognosis was deemed catastrophic but before a neurologist or neurosurgeon assessed and determined the absence of all brain stem reflexes as required by the Dutch brain death determination protocol (Scenario 3).

### Consultation by independent medical experts

**Figure 3:**
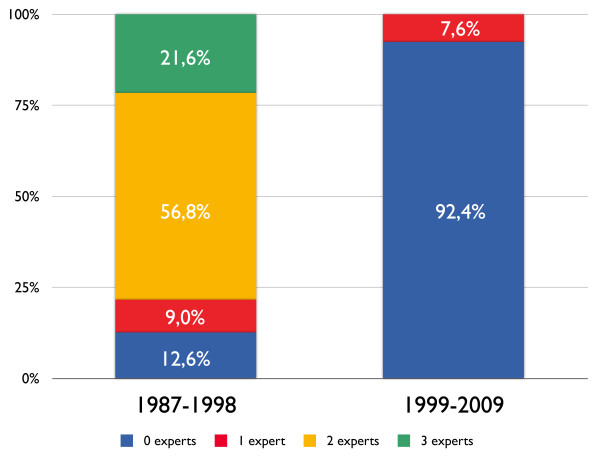
**The number of experts who independently reviewed the process of brain death determination**.

Before 1998, it was the custom in our ICU that two or more independent medical specialists from different departments (neurosurgery, neurology, surgery and/or internal medicine) confirmed the results of the completed brain death determination process. Before 1998, two or more experts reviewed the medical chart and obtained results of neurological examination and confirmatory tests, examined the patient and finally confirmed brain death determination, with 'no objection to organ donation' in 78.4% of the cases. This time-consuming but careful practise was gradually abolished after 1998 (Figure 3). We found no remaining record of an independent specialist confirming the brain death determination process after 2002. We must stress that there is a difference between the neurologist or neurosurgeon who assesses the absence of brainstem reflexes as described as step 1 in the Material and methods section of this article and the review of an independent specialist of the whole process.

### Influence of hand-written donor card and registration in donor register on consent rate

Before the introduction of the DR in 1998, patients could provide their consent for organ donation via a signed and dated hand-written donor card. In this period, we found a written donor card in 5.7% of the cases of effectuated organ donation. However, after the introduction of the DR, this form of consent was no longer tenable, and none was found after 1999. Before 1998, consent for organ donation was obtained from the next of kin of the deceased in the majority of the cases (94.3%, Table 2). Even in cases of a donor card, consent for organ donation was asked from the next of kin of a brain dead patient. In case of legitimate objections of the relatives, the physician was not constrained to proceed to organ donation. Following the introduction of the DR in the second period, a considerable fraction of the study population provided their own consent and preferences for organ donation through the DR. We found a positive registration in no less than 41.0% of the cases of effectuated organ donation.

**Table 2:** Demographics of effectuated brain-dead organ donors who gave consent for organ donation in the two study periods (*n *= 228)

	1987 to 1998 (*n *= 123)	1999 to 2009 (*n *= 105)	Total	*P*-value
**Age, yr (± SD)**	43.9 (15.2)	47.3 (15.7)	45.5 (15.5)	0.867
**Sex (M/F)**	71/52	47/58	118/110	0.051
**Consent no. (%)**				
**Family**	116 (94.3)	62 (59.0)	178 (78.1)	< 0.001
**Patient**	7 (5.7)	43 (41.0)	50 (21.9)	

## Discussion

### Timing of discussing organ donation with the relatives

We observed remarkable changes in the sequence of brain death determination in terms of the time at which organ donation was first discussed or consent for organ donation was first requested in relation to the sequence of brain death determination. A possible explanation for the changes in the sequence of the clinical and confirmatory tests with respect to the discussion of organ donation with the patients' relatives (Figure 2) is the introduction of the DR, which allowed the physician to consider the possibility of organ donation at an earlier time than was required before its introduction in 1998. If the physical condition of a patient made organ donation a plausible outcome, then the physician was expected to consult the DR to determine the registered wishes of the patient, leading to a possible decision about organ donation, which was made earlier in the whole process of organ donation.

In addition, DCD was reintroduced at our hospital in 1998. This required physicians to discuss organ donation and seek official consent before the withdrawal of life support in patients with catastrophic brain injury (but that were not (yet) brain-dead), and the practise may have extended to the pool of potential BD patients. A significant decline in DBD and an increase in DCD were observed in The Netherlands in the last 15 years [6]. The final explanation for the observed changes is that the physicians may have anticipated the possible adverse outcomes at an earlier stage due to the higher demand for ICU beds. If organ donation was refused and further treatment of the patient was judged to be futile, then withdrawal of life support was selected. The determination of an isoelectric EEG is not required for the decision to withdraw treatment in case of catastrophic brain injury. Continued treatment of a possible organ donor to keep vital organs in good condition is seen as a service for transplant medicine. The primary responsibility of the ICU physician is to provide good end-of-life care to patients with no hope of survival. Some will say that the continuation of futile treatment in the context of organ donation can be seen as the use of patients as a means rather than an end, but in case of a positive record in the DR and consent of the relatives of the patient who act on the conviction of the will of the patient, continued treatment can also be seen as an end and not as a means.

Refusal by relatives is the step in the donation process at which most donors are lost. Some studies have found that families are more willing to provide consent if they are given adequate information about brain death and the donation process [8,9]. An adequate understanding of brain death has been considered essential for obtaining donation consent. Rates of donation are higher when a timely brain death explanation is provided [10]. In a study from a European country with a high donor conversion rate (Spain), Andres *et al. *[11] state that relatives must correctly understand the brain death of the deceased before they are interviewed to request donation. The timing of the request is an important factor that can influence the rate of consent. The most important factor is a clear separation ('decoupling') in the time between the notification and acceptance of brain death and the request for organ donation. An analysis of a series of nine published reports by Simpkin *et al. *[9] suggested that there is an improved rate of consent when there is decoupling between notification and acceptance of brain death and the request for organ donation. Only one older study, by Niles and Mattice [12], found that consent was similar regardless of whether relatives were approached before (62%) or after (57%) death. Commonly mentioned reasons for the relatives to decline organ donation are concerns about disfigurement, emotionally overwhelmed feeling, inappropriate notification of brain death by physicians, and surprise at being asked for consent to donate [13,14]. Rodrigue *et al. *[10] found that for relatives who thought that the timing of the donation discussion was appropriate, 68% donated, whereas only 18% consented to donation if they considered the timing poor. Simpkin *et al. *[9] concluded after reviewing a series of published studies that the main and modifiable factors associated with consent or refusal for organ donation by relatives are perceived quality of care of the donor, understanding of brain death, timing of the request, the setting in which the request is made and the approach and skill of the individual making the request. The current standard in our hospital and many other Dutch hospitals of informing the patients' relatives and asking consent for organ donation prior to formal brain death determination does not address the above-mentioned issues. In The Netherlands the refusal rates by relatives approached for a family member's organ donation were 65% in 2005, 71% in 2006, 59% in 2007 and 69% in 2008 [15]. Taking the literature on this subject into account, it is possible that we shoot ourselves in the foot on this subject.

Besides the aforementioned study by Niles and Mattice [12], we were unable to find any evidence that requesting organ donation before or after death has a positive or negative effect on the consent rate. However, the results of many published studies on the effect of decoupling the announcement of brain death and the request for organ donation, and the positive impact of a clear explanation of brain death, suggest that a pre-BD request may have a negative effect on the relatives' consent rate. The continuing gap between the need and demand for organs for transplantation forces us to perform such an emotional and delicate process as brain death determination and notification with the highest ethical and medical standards.

Consequently, and based on the results of many studies, we believe that consent for organ donation should only be requested and obtained after the full formal brain death determination.

### Consultation by independent medical experts

The number of medical experts who reviewed the whole process of brain death determination declined to zero after 2002. It was not compulsory but was permitted in the national brain death protocol to invite an independent medical expert to review the completed BD determination process. This was an extra safeguard in our hospital that nothing be overlooked or missed that could make the conclusion that a patient was brain dead invalid. Why this practise is not continued remains unclear. It was comforting for relatives that an elusive state such as brain death be confirmed by independent medical specialists. Certain states in the US still require independent confirmation by another physician of the declaration of brain death [16]. In European countries, there are no requirements of an independent review of the brain death process, although the number of physicians that should be involved varies per country from zero to four.

### Influence of hand-written donor card and registration in donor register on consent rate

The introduction of the DR formalised the process of organ donation. One of the aims of the DR was to provide individuals with the option of registering their preferences. Physicians could easily locate the registration of the potential organ donor in the DR and act according to the patient's wishes. Our data suggest that the DR has been beneficial over the past decade. Families are no longer obligated to make an *ad hoc *decision about organ donation if a positive (consent) or negative (objection) registration is available. A potential disadvantage is that families seem to be more reluctant to consent to organ donation if the patient is not registered. If someone believes that being an organ donor is important to him or her, then registration would be expected. Although we have no scientific proof on this matter, this is the common experience of health care professionals in The Netherlands. The mean refusal rate in our hospital in the first study period was 31% but increased in the second period to 45% (unpublished data, Erasmus MC Rotterdam). The reason for this rise in refusal rate is complex and difficult to determine, but reluctance of relatives in cases of non-registration could be a major factor.

According to the latest report (August 2011) of the Dutch DR [17], 33.5% of the Dutch population had registered their decision for organ donation, amongst whom 58.8% stated that they wished to donate one or more organs, whereas 28.7% refused to donate, 10.7% left the decision to donate to their relatives and 1.8% to an appointed person. Debate has raged in The Netherlands about the advantages and disadvantages of the opt-in and opt-out systems [18].

### Study limitations

Our study has some limitations. First, the study was performed in a single centre, and data were obtained through a retrospective review of medical charts. However, our hospital is one of the largest university hospitals in The Netherlands, with a large neurosurgical service area, and hence, the results may be exemplary for other large hospitals in the country. As with all observational studies, some cases may have been missed in a non-random manner. Due to the retrospective nature of our study, we could not obtain the charts of all potential brain-dead organ donors or of all brain-dead patients who did not donate organs because there was no formal listing of this group of patients. However, the data obtained from this study are valuable for the study of the influence of the DR on the moment of consent. Further studies should be conducted to determine whether requesting consent for organ donation before neurological examination or before confirmatory testing in possible BD patients has a positive, neutral or negative influence on the consent rate.

## Conclusions

The sequence of brain death determination and the time at which organ donation is first discussed with relatives have changed over time. Currently, in most cases, the issue of organ donation is first discussed after the clinical-neurological assessment as described in the brain death determination protocol but before the required confirmatory tests. Possible causes of this change are the introduction of the DR, the reintroduction of donation after cardiac death and other logistical factors. It is unclear whether the observed shift contributed to the high refusal rate in The Netherlands and the increase in family refusal in our hospital in the second studied period. Taking the published literature on this subject into account, it is possible that this may have a counterproductive effect on the matter. After the introduction of a national DR, donation by patient consent has increased from 5.7% to 41.0%.

## Key messages

• Over the past 15 years in The Netherlands, there has been a decline in donation after brain death from 89% to 58%, while donation after circulatory death increased from 11% to 42% in the same period.

• After introduction of the Donor Register in the Netherlands, the rate of organ donation by patient consent in our hospital increased from 5.7% to 41.0%.

• Before 2002, independent medical experts reviewed and confirmed the outcome of the whole process of brain death determination of a potential brain-dead organ donor and documented their findings in the medical chart. After 2002, this careful procedure is no longer conducted.

• The time at which organ donation is first discussed with relatives has changed over time in our hospital. Initially, between 1987 and 1998, organ donation was mentioned for the first time after completion of ancillary tests conforming to the national brain death protocol. After 1998, in most cases, organ donation was discussed after determination of loss of consciousness and the absence of brainstem reflexes but before completion of the confirmatory tests.

## Abbreviations

BD: brain death; DCD: donation after cardiac death; DBD: donation after brain death; DR: Donor Register; DODA: Dutch Organ Donation Act; ICH: intracerebral haemorrhage; SAH: subarachnoid haemorrhage; TBI: traumatic brain injury

## Competing interests

The authors declare that they have no competing interests.

## Authors' contributions

YG conceived and designed the study, retrieved the necessary data and wrote the first draft of the manuscript. EK conceived and designed the study, retrieved the necessary data, coordinated the study and made a substantial contribution to the draft and the later versions of the manuscript. JB participated in the study design and helped in drafting the manuscript. HL performed the statistical analysis and helped in drafting the manuscript. JIJ and MJ helped in drafting the manuscript. All authors read and approved the final manuscript for publication.
